# Antisense oligonucleotide treatment in a preterm infant with early-onset SCN2A developmental and epileptic encephalopathy

**DOI:** 10.1038/s41591-025-03656-0

**Published:** 2025-04-22

**Authors:** Matias Wagner, Géza Berecki, Walid Fazeli, Claudia Nussbaum, Andreas W. Flemmer, Silvana Frizzo, Farina Heer, Florian Heinen, Robert Horton, Henry Jacotin, William Motel, Brian Spar, Christoph Klein, Corinna Siegel, Christoph Hübener, Sophia Stöcklein, Marco Paolini, Martin Staudt, Moritz Tacke, Markus Wolff, Steven Petrou, Marcio Souza, Ingo Borggraefe

**Affiliations:** 1 Division of Pediatric Neurology and Developmental Medicine, Department of Pediatrics and Comprehensive Epilepsy Center, Munich University Center for Children with Medical and Developmental Complexity – MUC iSPZ Hauner, Dr. v. Hauner Children’s Hospital, Ludwig Maximilians University Hospital, Munich, Germany; 2 Institute of Human Genetics, School of Medicine and Health, Technical University of Munich, Munich, Germany; 3 Institute for Neurogenomics, Helmholtz Centre Munich, German Research Center for Health and Environment, Munich, Germany; 4 Ion Channels and Human Diseases Group, The Florey Institute of Neuroscience and Mental Health, University of Melbourne, Parkville, Victoria Australia; 5 Department of Pediatric Neurology, Children’s Hospital, University Hospital Bonn, Bonn, Germany; 6 Division of Neonatology, Dr. v. Hauner Children’s Hospital, Ludwig Maximilians University Hospital, Munich, Germany; 7 Praxis Precision Medicines, Boston, MA USA; 8 Department of Pediatrics, Dr. v. Hauner Children’s Hospital, Ludwig Maximilians University Hospital, Munich, Germany; 9 MVZ Martinsried GmbH, Martinsried, Germany; 10 Department of Obstetrics and Gynecology, Ludwig Maximilians University Hospital, Munich, Germany; 11 Department of Radiology, Ludwig Maximilians University Hospital, Munich, Germany; 12 Department of Pediatric Palliative Care, Ludwig Maximilians University Hospital, Munich, Germany; 13 Swiss Epilepsy Center, Klinik Lengg AG, Zurich, Switzerland

**Keywords:** Epilepsy, Drug development, Genetics research, Drug safety

## Abstract

Early-onset *SCN2A* developmental and epileptic encephalopathy is caused by *SCN2A* gain-of-function variants. Here we describe the clinical experience with intrathecally administered elsunersen, a gapmer antisense oligonucleotide targeting *SCN2A*, in a female preterm infant with early-onset *SCN2A* developmental and epileptic encephalopathy, in an expanded access program. Before elsunersen treatement, the patient was in status epilepticus for 7 weeks with a seizure frequency of 20–25 per hour. Voltage-clamp experiments confirmed impaired channel inactivation and increased persistent current consistent with a gain-of-function mechanism. Elsunersen treatment demonstrated a favorable safety profile with no severe or serious adverse events reported after 19 intrathecal administrations over 20 months. After administration in combination with sodium channel blockers, status epilepticus was interrupted intermittently and ultimately ceased after continued dosing. A >60% reduction in seizure frequency corresponding to five to seven seizures per hour was observed, which has been sustained during follow-up until the age of 22 months. These data provide preliminary insights on the safety and efficacy of elsunersen in a preterm infant. Additional investigation on the benefits of elsunersen in clinical trials is warranted.

## Abstract (web summary)

Administration of an antisense oligonuceotide in a preterm infant with severe early-onset SCN2A developmental and epileptic encephalopathy was well tolerated and led to a 60% decrease in seizure frequency.

## Main

*SCN2A*-associated developmental and epileptic encephalopathy (*SCN2A*-DEE, Mendelian Inheritance in Man (MIM): #613721) is a rare monogenetic disorder characterized by early-onset (<3 months of age) seizures and profound developmental impairment^1^. The *SCN2A* gene encodes the sodium channel Na_v_1.2, which is predominantly expressed in excitatory neurons of the central nervous system^2^. Variants in *SCN2A* that cause loss of function are associated with an encephalopathy characterized by onset of seizures at typically more than 3 months of age or with autistic features without seizures. Variants that cause biophysical gain-of-function (GoF) changes can present with a distinct spectrum of disorders ranging from self-limited familial neonatal–infantile seizures (MIM: #607745)^3^ and episodic ataxia type 9 (MIM: #618924)^4^ to a severe form of *SCN2A*-DEE (MIM: #613721) where seizures are often difficult to control with conventional antiseizure medications^1^. While sodium channel blockers were shown to reduce seizure frequency in cases with GoF variants, motor skills, language acquisition and cognitive abilities may be substantially impaired^5^. Antisense oligonucleotide (ASO) therapies have emerged as a promising class of therapeutic interventions for a range of genetic disorders^6^. It has been shown in vitro and in a *Scn2a* GoF mouse model that treatment with a mouse-specific gapmer ASO could safely reduce mRNA levels and seizure burden^7^, paving the way for clinical development in patients with *SCN2A* GoF-associated DEE. *SCN2A* is highly intolerant against both heterozygous loss of function (haploinsufficiency) and dosage increase (triplosensitivity) (PMID: 28176757), suggesting a small therapeutic window of medical knockdown strategies. In this context, *SCN2A*-DEE could be treated using gapmer ASOs either in an allele-selective fashion where the ASO targets the causative variant directly or via a heterozygous polymorphism located on the same allele or unspecifically where both the mutant and the wild-type (WT) allele are targeted. The latter approach comes with a higher danger of overdosing, whereas allele-selective approaches come with limited targeted regions often resulting in lower efficacy and increased toxicity.

Here, we describe a female patient with early-onset DEE caused by a GoF variant in *SCN2A* treated with multiple intrathecal administrations of elsunersen (PRAX-222), an allele-nonselective gapmer ASO targeting *SCN2A* mRNA.

**Extended Data Fig. 1:**
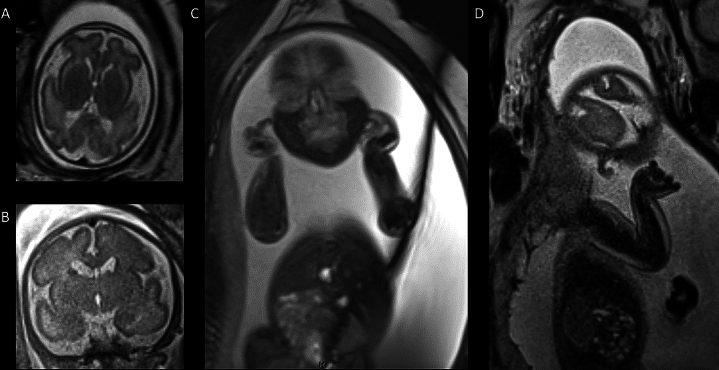
Prenatal fetal MRI showing arthrogryposis and large basal ganglia. **A** and **B** Prenatal fetal MRI at 29 + 2 weeks of gestational age shows prominent basal ganglia but otherwise normal gyration and myelination. **C** and **D** Imaging of the extremities shows hyperextended wrists and fisting with crossing of fingers.

The patient is a preterm infant born at 29 + 4 weeks of gestational age with a birth weight of 1,400 g. Polyhydramnios as well as constantly flexed arms with hyperextended wrists and fisting of the hands had been noticed on ultrasound examination at 27 weeks of gestation. Fetal magnetic resonance imaging (MRI) confirmed the ultrasound findings (suspected arthrogryposis; Extended Data Fig. 1). Prenatal parent–child trio exome sequencing detected the de novo variant c.3986C>A in *SCN2A* (NM_001040142.2) causing an amino acid substitution p.Ala1329Asp (p.A1329D) in the α-helical intracellular linker between transmembrane segments S4 and S5 of domain III. At postnatal day 1, the patient presented with status epilepticus (SE as defined by ref. ^8^) clinically (mostly sequential automotor and tonic seizures) and confirmed by amplitude-integrated electroencephalography (aEEG; Supplementary Fig. 1a). Subsequent antiseizure medication included phenobarbital, levetiracetam, phenytoin, vitamin B6, lacosamide, oxcarbazepine, midazolam (continuous infusion), carbamazepine and lidocaine that did not terminate the SE. Phenytoin interrupted SE transiently (maximal interruption of SE of about 20 min; Supplementary Fig. 1b), but SE reoccurred despite phenytoin serum levels >40 µg ml^−1^ (Supplementary Fig. 1c).

Postnatal brain MRI (Supplementary Fig. 2) revealed callosal hypoplasia, bilateral focal T2-weighted medullary hyperintensities in the frontotemporal region as well as rather slender impinging thalami in addition to prominent, extended lateral ventricles. Considering the severity of the course, new therapeutic approaches were warranted.

**Fig. 1:**
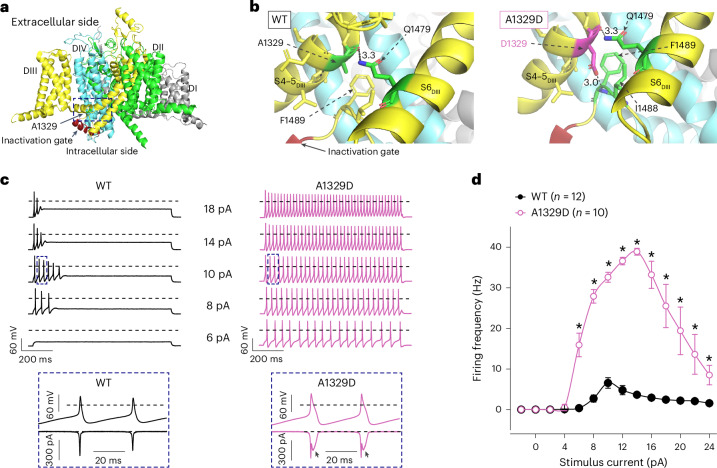
Location and functional impact of the A1329D Na_v_1.2 channel mutation. **a**, Side view of the 3D structure of Na_v_1.2 highlighting the A1329 residue (arrow) residing in the α-helical intracellular linker between transmembrane segments S4 and S5 of domain III (S4–5_DIII_) (dashed boxed area). **b**, Zoomed-in views highlighting the amino acid residues before (left) and after (right) in silico mutagenesis. All residues shown in stick representation are within 5 Å of the A1329 or D1329 residues. In the mutant channel, D1329 forms polar interactions with Q1479 and F1489. The D1329–F1489 interaction is likely to affect the binding of the I1488–F1489–M1490 (IFM) inactivation motif to its receptor site, resulting in delayed inactivation and persistent current. **c**, Representative action potential firing of the axon initial segment hybrid compartment model incorporating WT or A1329D Na_v_1.2 channel variant in response to stepwise increase in the stimulus current amplitude (6, 8, 10, 14 or 18 pA) in DAPC experiments. The dashed lines indicate the 0 mV level. Action potentials (bottom) on an expanded timescale (upward deflections), corresponding to action potentials enclosed in dashed boxes elicited by 10 pA stimulus current (top), and associated scaled input sodium currents (downward deflections). Note the apparent increase of the action potential width in the presence of A1329D and the associated increase of the inward sodium current component (arrows) compared with WT. The dashed lines indicate the 0 mV and 0 pA levels, respectively. In all experiments, the virtual sodium conductance of the axon initial segment (AIS) model was set to zero (gNa_v_1.6 = 0), whereas the virtual K_v_ channel conductance was set to 2 (twice the original gK_v_). In all DAPC experiments, the original (nonscaled) external sodium current *I*_Nav1.2_, the scaled external *I*_Nav1.2_, the membrane potential (*V*_m_), and gK_v_ were simultaneously recorded. **d**, Input–output relationships for WT and A1329D variants. Data are represented as mean ± s.e.m.; *n*, the number of experiments between parentheses. The asterisks indicate statistically significant differences in the presence of A1329D relative to WT (*P < 0.05, two-way ANOVA, followed by Dunnett’s post-hoc test; see individual *P* values in Supplementary Table 3).

**Extended Data Fig. 2:**
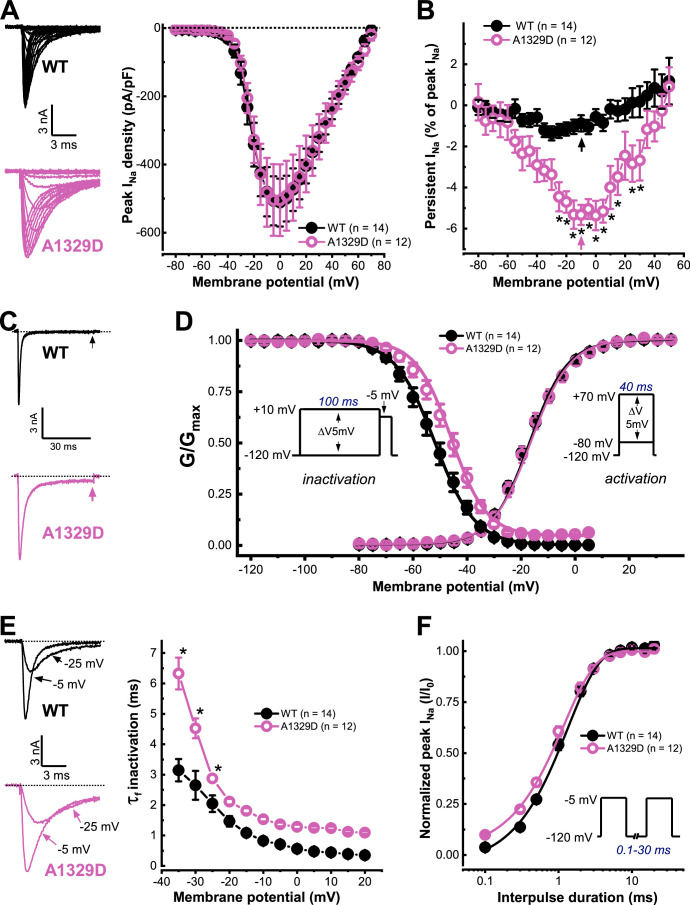
Biophysical properties of the A1329D Nav1.2 channel mutation as determined by voltage clamp experiments. **A** Peak sodium current (I_Na_) density-voltage relationships. Currents were elicited from a holding potential of −120 mV by depolarizing voltage steps of 40 ms duration in 5 mV increment in the voltage range between −80 and +70 mV (voltage protocol shown in *right* inset in D). Left: representative I_Na_ traces in the voltage range between −80 and +20 mV; note that only the first 10-ms of the traces are shown. **B** Persistent inward I_Na_-voltage relationships. Persistent current amplitude was determined as the inward current amplitude 40 ms after depolarization (arrows). Dotted lines indicate 0-pA level. **C** representative WT and A1329D I_Na_ traces elicited by −10 mV depolarizations. **D** Activation was determined from current-voltage relationships shown in A, whereas inactivation was determined from a holding potential of −120 mV, using 100-ms conditioning steps (between −120 and +10 mV) followed by a 20-ms test pulse to −5 mV to reveal the available current (arrow), at 0.1 Hz (voltage protocol shown in *left* inset). Voltage dependence of activation and inactivation, respectively, were obtained by measuring macroscopic currents and fitting the observed voltage dependence of the normalised conductance (G/Gmax) to a Boltzmann equation as follows: $\frac{{\rm{G}}}{{{\rm{G}}}_{\max }}=\frac{1}{[1+{{\rm{e}}}^{({\rm{V}}-{{\rm{V}}}_{0.5})/{\rm{k}}}]}$, where V is the membrane voltage, V_0.5_ represents the voltage for half-maximal activation or inactivation (V_0.5,act_ or V_0.5,inact_, respectively), and k is the slope factor. Conductance (G) was calculated using the equation G = I/(V - V_rev_), where V_rev_ represents the Na⁺ reversal potential. Normalized conductance values (G/G_max_) were plotted against the membrane potential to generate activation curves. **E** Dependence of the time course of I_Na_ inactivation on the membrane potential. Left: Representative WT and A1329D I_Na_ traces elicited by −25 and −5 mV voltages. Note the slower time course of A1329D I_Na_ relative to WT. The fast time constants (τ_f_) were obtained by fitting a double-exponential equation to the inactivating segment of individual I_Na_ traces as follows: $\frac{{\rm{I}}}{{{\rm{I}}}_{\max }}={{\rm{A}}}_{{\rm{f}}}{{\rm{e}}}^{-{\rm{t}}/{{\rm{\tau }}}_{{\rm{f}}}}+{{\rm{A}}}_{{\rm{s}}}{{\rm{e}}}^{-{\rm{t}}/{{\rm{\tau }}}_{{\rm{s}}}}$, where t is time, A_f_ and A_s_ are the fractions of the fast and slow inactivation components, and τ_f_ and τ_s_ are the time constants of the fast and slow inactivating components, respectively. **F** Recovery from fast inactivation was evaluated using a paired-pulse voltage protocol from a holding potential of −120 mV (inset protocol). The first pulse inactivated the channels, followed by a second pulse to measure the fraction of current that recovered from inactivation after inter-pulse intervals of increasing duration. The time constants of recovery (τ) were determined by fitting the data with a single exponential function, as follows: $\frac{{\rm{I}}}{{{\rm{I}}}_{\max }}=1-{{\rm{e}}}^{-{\rm{t}}/{\rm{\tau }}},$ where t is the time between the P1 and P2 test pulses. Data are represented as mean ± SEM; n, the number of experiments are shown between parentheses. Asterisks indicate statistically significant differences in the presence of A1329D relative to WT (*P < 0.05). The parameters of the fits and the results of the statistical evaluation are displayed in Supplementary Table 1.

To assess a possible amenability to treatment with elsunersen, we performed in silico three-dimensional (3D) modeling as well as electrophysiological studies. Modeling suggested that the variant impairs the binding of the inactivation motif to its receptor (Fig. 1a,b). Voltage-clamp experiments confirmed structural modeling predictions that in the mutant channel the Asp1329 residue interferes with the binding of the inactivation motif, leading to GoF via impaired inactivation and increased persistent current (Extended Data Fig. 2 and Supplementary Tables 1 and 3). Furthermore, dynamic action potential clamp (DAPC) experiments showed a strongly increased action potential firing rate in response to increasing step current stimuli and a significantly reduced rheobase compared with WT (Fig. 1c,d) consistent with in vitro findings in other DEE cases^9^.

**Fig. 2:**
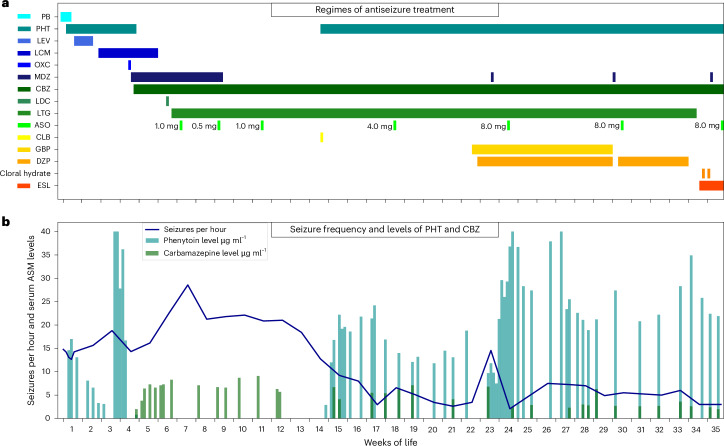
The patient clinical course after introduction of the elsunersen treatment regimen and effects on seizures. **a**, The antiseizure medication (ASM) regimen including high-dose sodium channel blockers and introduction of the elsunersen dosing regimen. **b**, The associated reduction in seizure frequency. A total of seven elsunersen (intrathecal) doses were administered between 13 March 2023 and 29 September 2023 (30.5 mg total). PB, phenobarbital; PHT, phenytoin; LEV, levetiracetam; LCM, lacosamide; OXC, oxcarbazepine; MDZ, midazolam; CBZ, carbamazepine; LDC, lidocaine; LTG, lamotrigine; ASO, antisense oligonucleotide, here elsunersen; CLB, clobazam; GBP, gabapentin; DZP, diazepam; ESL, eslicarbazepine. Corresponding aEEG traces are displayed in Supplementary Fig. 1a–g.

At the age of 7 weeks, we started the intrathecal application of elsunersen after all standard antiseizure medication failed to terminate the SE for an initial observational period until the age of 8 months. Eight days after the first administration (for concomitant drugs, see Fig. 2a), SE was intermittently interrupted and subsequently terminated after subsequent doses. (Fig. 2 and Supplementary Fig. 1d–g). After reaching 12 weeks of age and receiving a cumulative dose of 2.5 mg of elsunersen across three administrations, seizure frequency further declined in combination with phenytoin that has been reintroduced (Fig. 2 and Supplementary Fig. 1d–g). Mean seizure frequency per hour within the first 12 weeks of the observation period was 19.7 (±4.2; range 13.8–28.6) and decreased by 67% compared with the remaining observation period from week 13 to 35 (6.6 ± 4.3; range 2.1–18.4). The increase of seizure frequency in week 23 was attributed to urosepsis and possibly related to a transient decline of phenytoin plasma levels. The parents noted a reduction in seizure severity 3–4 weeks after the first elsunersen application. After the initial three doses described above, an additional four doses were administered, resulting in a cumulative dosage of 30.5 mg elsunersen across all seven doses (Fig. 2a).

**Extended Data Fig. 3:**
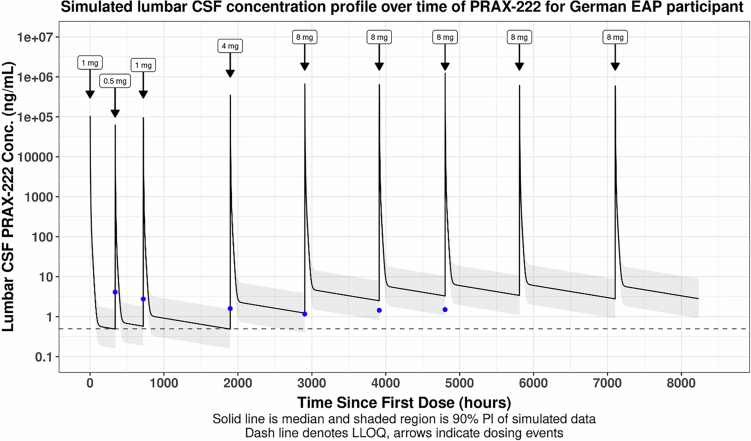
Simulated concentration-time profile of PRAX-222 in lumbar CSF. Simulated median (90% PI) PRAX-222 concentration-time profile in lumbar CSF following IT-L administration of titrated PRAX-222 dose amounts to the single EAP participant overlaid upon the observed data. Conc.: concentration; CSF: cerebrospinal fluid; EAP: Emergency Access Program; IT-L: intrathecal lumbar; LLOQ: lower limit of quantification; PI: prediction interval.

**Extended Data Fig. 4:**
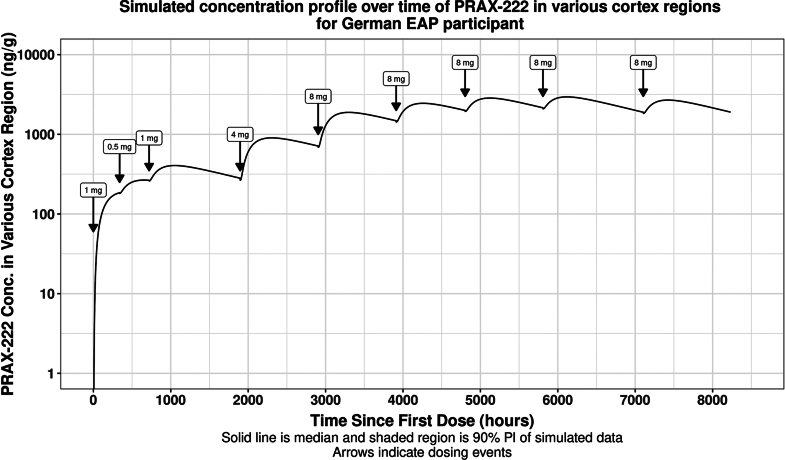
Simulated concentration-time profile of PRAX-222 in the cerebral cortex. Simulated median PRAX-222 concentration-time profile in the frontal/motor/temporal cortex following IT-L administration of titrated PRAX-222 dose amounts to the single EAP participant Conc.: concentration; CSF: cerebrospinal fluid; EAP: Emergency Access Program; IT-L: intrathecal lumbar; LLOQ: lower limit of quantification; PI: prediction interval.

The parents reported that seizure frequency and severity increased approximately 4–6 weeks after each administration. Hence, we modeled the concentration–time profiles of elsunersen in lumbar cerebrospinal fluid (CSF) as well as in the cerebral cortex based on the concentrations in CSF at the time of drug administration (after nine dosages). Indeed, elsunersen showed little accumulation and moderate retention time in the brain (Extended Data Figs. 3 and 4). These data suggest that it is necessary to administer elsunersen at intervals of 4–6 weeks. This observation is particularly relevant for *n*-of-1 and small-cohort (*n*-of-few) clinical trials with ASOs where pharmacokinetic data may be limited.

Neurodevelopmental assessment during a period of SE was difficult. The patient displayed severe hypotonia between the seizures. Motor reactions were primarily pain related during the first weeks of life. Ophthalmologic investigation revealed atrophy of the optical papillae at the age of two and a half months. After the status terminated and seizure frequency improved, neurodevelopment was still severely affected during the observation period and minimal milestones such as swallowing were achieved. At the end of the initial observational period at the chronological age of 8 months, the patient presented with severe hypotonia, indicated by an inability to control head movements, yet retained the capacity to swallow despite the necessity of a feeding tube. The patient exhibited nonspecific motor responses to auditory stimuli and demonstrated minimal reactions to bright visual stimuli, indicative of blindness.

**Extended Data Fig. 5:**
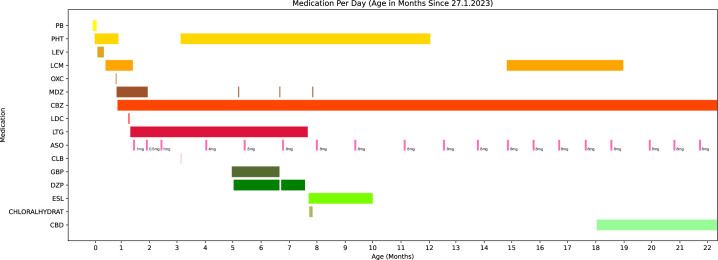
Treatment regimen covering the extended observational period. A total of 94 mg elsunersen (intrathecal) doses were administered between until the age of 21 months. Abbreviations: CBD: cannabidiol, ESL: eslicarbazepine DZP: diazepam, GBP: gabapentin, CLB: clobazam, ASO: antisense oligonucleotide, here elsunersen, LTG: lamotrigine, LDC: lidocaine, CBZ: carbamazepine, MDZ: midazolam, OXC: oxcarbazepine, LCM: lacosamide, LEV: levetiracetam, PHT: phenytoin, PB: phenobarbital.

As the treatment with elsunersen seemed to result in a control of seizure frequency, treatment was continued after discussions with the parents. Within the extended observational period until the age of 22 months, the patient received further ten dosages of 8 mg elsunersen on a monthly basis. Seizure frequency remained stable at no more than five seizures per hour. This also maintained after tapering phenytoin at the age of 14 months (Supplementary Table 2). The antiseizure therapy regimen during the extended observational period is shown in Extended Data Fig. 5. No safety concerns related to the drug were observed during this period. Neurodevelopmental assessment and monthly sleep–wake EEGs (Supplementary Fig. 3a–o) revealed no worsening. However, the patient remained severely impaired (that is, poor head control and severe muscular hypotonia) at the age of 18 months, underscoring the need for future studies to explore whether elsunersen can contribute to neurodevelopment improvements beyond seizure control. However, we hypothesize that the severe neurodevelopmental outcome in this case is related to initial severity of disease presentation and the extrapolated burden of approximately 60,000 seizures over an 8-month period.

At the last follow-up at the age of 22 months, the parents reported a seizure frequency of approximately five to seven per hour and is continuously dosed with 8 mg elsunersen on a monthly basis. An ictal EEG of the patient at the age of 22 months is shown in Supplementary Fig. 4.

**Extended Data Fig. 6:**
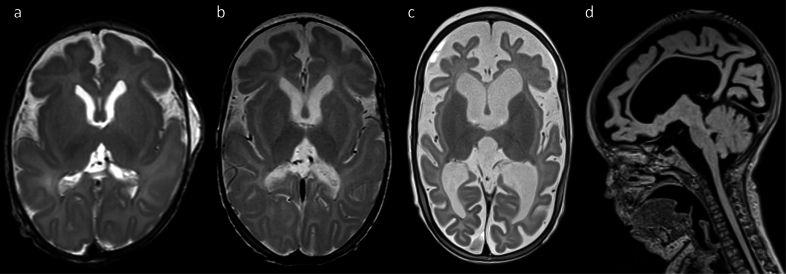
cMRIs at the age of 5 weeks (**a**), 17 weeks (**b**) and 36 weeks (**c** and **d**). cMRI shows progressive symmetrical atrophy of the cerebral cortex sparing the basal ganglia, the thalamus and the cerebellum.

Vital signs and blood tests did not reveal any persistent abnormalities that could be directly attributed to the administration of elsunersen within the observed timeframe. Similarly, electrocardiogram and echocardiography examinations showed no drug adverse effects. Notably, there was no observation of hydrocephalus, a potential ASO class effect^10^ Sequential brain MRI scans conducted at 5, 17 and 36 weeks of age demonstrated progressive global atrophy of the cerebral cortex while sparing the basal ganglia, thalami and cerebellum (Extended Data Fig. 6a–d). These findings are probably attributable to the underlying disease, as similar patterns of atrophy have been documented in patients with GoF *SCN2A*-DEE^11–13^.

The observed temporal correlation between the intrathecal administration of elsunersen in combination with sodium channel blockers and a notable seizure reduction, including the cessation of SE in addition to the reported increase in seizure frequency and severity 4–6 weeks after each administration supported by pharmakokinetic modeling, suggests a causal association. Given that the data are based on one single patient, establishment of a definitive causal link will require further validation through formal clinical trials. The rapid onset of seizure reduction, noticeable as early as 8 days after the initial administration of elsunersen, and continued improvement after administering a cumulative dose of 2.5 mg over 5 weeks aligns with biological models. This timing is consistent with known turnover rates of *SCN2A* mRNA and Na_v_1.2 sodium channels on the plasma membrane^14^, supporting the plausibility of elsunersen’s mechanism of action in this context.

In *SCN2A* early-onset DEE, optimal seizure control may be attained through an adjunctive approach combining elsunersen for direct therapy of the underlying genetic etiology and sodium channel blockers for fine-tuning neuronal excitability. In conclusion, our findings support elsunersen’s potential as a disease-modifying therapy for early-onset GoF *SCN2A*-DEE.

## Methods

### Patient eligibility

The institutional clinical ethics committee (LMU Munich) reviewed the case and concluded that the criteria of a treatment with elsunersen in a named patient setting (‘individueller Heilversuch’, Declaration of Helsinki Revision 2013, § 37) were fulfilled. Elsunersen was provided within an expanded access program by Praxis Precision Medicines and not specifically developed for the treatment of this patient. The initial treatment and observational period were chosen until the age of 8 months with regular safety assessments (at least once before ASO application and on a regular basis after the applications) including electrocardiogram, echocardiography, vitals, blood examinations (whole blood cell count, blood urea nitrogen, creatinine, liver enzymes, serum electrolytes and coagulation parameters, and blood gas analysis), clinical examination, CSF (cell count, protein), cranial ultrasound and MRI. The further dosing strategy of elsunersen was determined depending on the clinical response and the occurrence of possible side effects after each administration. Within the extended observational period until the age of 14 months, safety examinations were conducted only at the time of ASO applications and limited to EEG and blood sampling (monthly basis). Written informed consent of both parents for the treatment as well as for the public distribution of observational data and findings was obtained before ASO administration.

aEEG and EEG analysis was performed by two board-certified (German Society for Clinical Neurophysiology) pediatric neurologists (I.B. and M.T.)

### Na_v_1.2 channel clones, cell culture and transfection

The c.3986C>A (p.A1329D) mutation was introduced into the open reading frame of SCN2A within the pcDNA3.1(+) plasmid vector, which incorporates the adult isoform of the human *SCN2A* cDNA. The mutation was generated using QuikChange site-directed mutagenesis (Agilent Technologies) with forward and reverse primers GAATGAGGGTTGTTGTAAATGATCTTTTAGGAGCCATTCCATC and GATGGAATGGCTCCTAAAAGATCATTTACAACAACCCTCATTC, respectively, and verified by Sanger sequencing (Australian Genome Research Facility). Chinese hamster ovary cells were cultured in Dulbecco’s modified Eagle medium:nutrient mixture F-12 (Thermo Fisher Scientific) supplemented with 10% (v/v) fetal bovine serum (Thermo Fisher Scientific) and 50 IU ml^−1^ penicillin (Thermo Fisher Scientific) in 25-cm^2^ flasks (BD Biosciences) at 37 °C with 5% CO_2_. At ~80 % confluency, the cells were transiently co-transfected with WT or mutant pcDNA3.1(+)-Na_v_1.2 (5 μg) construct and enhanced green fluorescent protein (1 μg, Clontech), using Lipofectamine 3000 Reagent (Thermo Fisher Scientific). Then, 3–4 days after transfection, the cells were detached using TrypLE Express Reagent (Thermo Fisher Scientific), plated on 13-mm-diameter glass coverslips (Menzel-Gläser, Thermo Fisher Scientific) and used for electrophysiological recordings^9^.

### Electrophysiology

Voltage-clamp recordings were performed at room temperature (23.0 ± 0.5 °C). The cells were superfused at a rate of ~0.2 ml min^−1^ with extracellular solution containing 145 mM NaCl, 5 mM CsCl, 2 mM CaCl_2_, 1 mM MgCl_2_, 5 mM glucose, 5 mM sucrose and 10 mM HEPES (pH 7.4 with NaOH) and intracellular solution containing 5 mM CsCl, 120 mM CsF, 10 mM NaCl, 11 mM EGTA, 1 mM CaCl_2_, 1 mM MgCl_2_, 2 mM Na_2_ATP and 10 mM HEPES (pH 7.3 with CsOH). Whole-cell sodium currents (*I*_Na_) were recorded using an Axopatch 200B amplifier (Molecular Devices) controlled by a pCLAMP 10/DigiData 1440 acquisition system (Molecular Devices). The current density, voltage dependence of *I*_Na_ activation and inactivation, recovery from fast inactivation, and the *I*_Na_ kinetics were determined using the experimental protocols and equations described in the legend of Extended Data Fig. 2. Real-time DAPC recordings were performed by introducing heterologously expressed and scaled WT or A1329D *I*_Na_ into a biophysically realistic axon initial segment model^9,15^. The DAPC experimental settings are detailed in the legend of Fig. 1.

### 3D modeling

For 3D modeling of the A1329D variant, we used the experimentally solved structure of human Na_v_1.2 channel (PDB ID 6J8E) and evaluated the impact of the mutation on the structure of Na_v_1.2 (ref. ^16^). Visualizations were generated using PyMOL software (version 1.8.5.0).

### Drug design

**Extended Data Fig. 7:**
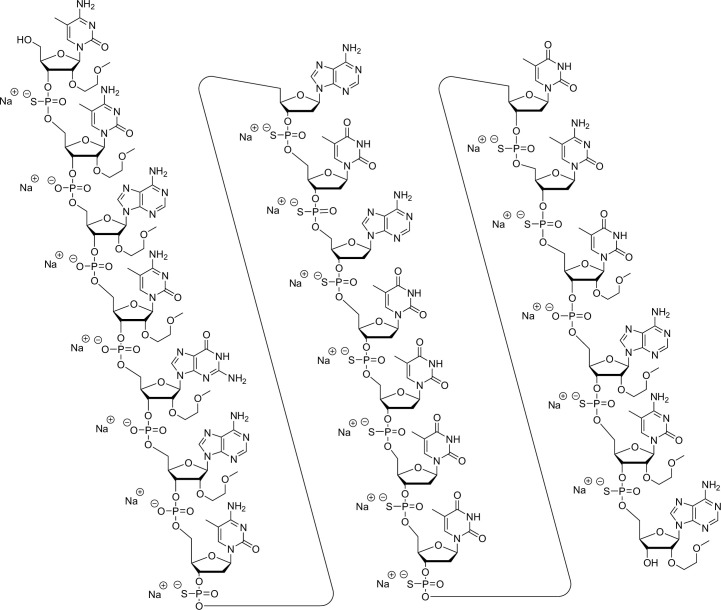
Chemical Structure of PRAX-222 sodium (elsunersen sodium). PRAX-222 sodium (elsunersen sodium) is a 20-mer single stranded 2’-O-methoxyethyl (MOE) gapmer oligonucleotide with mixed phosphorothioate (n = 13) and phosphodiester (n = 6) backbone linkages. The nucleotide sequence is 5’-CCACGACATATTTTTCTACA-3’.

PRAX-222 sodium (elsunersen sodium) is a 20-mer single-stranded 2′-O-methoxyethyl gapmer oligonucleotide with mixed phosphorothioate and phosphodiester backbone linkages. The nucleotide sequence is 5′-CCACGACATATTTTTCTACA-3′ and registered under CAS# 2755996-85-9. The manufacturing processes for elsunersen follow current Good Manufacturing Practices regulations, and the drug product process follows all requirements for the manufacture of sterile medicinal products. Its chemical structure is shown in Extended Data Fig. 7.

### Reporting summary

Further information on research design is available in the Nature Portfolio Reporting Summary linked to this article.

## Online content

Any methods, additional references, Nature Portfolio reporting summaries, source data, extended data, supplementary information, acknowledgements, peer review information; details of author contributions and competing interests; and statements of data and code availability are available at 10.1038/s41591-025-03656-0.

## Supplementary information

Supplementary Information Supplementary Figs. 1–4 and Tables 1–3.

Reporting Summary

## Data Availability

All data not included in this Brief Communication or its Supplementary Information may be requested for appropriate use from the corresponding author (in a pseunonymized way for clinical data if in line with the consents provided). Requests will be reviewed within 3 weeks.
