# Supplementary material for: Antisense oligonucleotide treatment in a preterm infant with early-onset SCN2A developmental and epileptic encephalopathy
Source: Nat Med. 2025 Apr 22;31(7):2174–8. doi: 10.1038/s41591-025-03656-0 (PMC12283366; doi:10.1038/s41591-025-03656-0)
Supplement: Supplementary file 1 — Supplementary Figs. 1–4 and Tables 1–3. [file 41591_2025_3656_MOESM1_ESM.pdf]

nature medicine

Supplementary information https://doi.org/10.1038/s41591-025-03656-0

Antisense oligonucleotide treatment in a preterm infant with early-onset SCN2A developmental and epileptic encephalopathy

In the format provided by the authors and unedited

Antisense Oligonucleotide Treatment in a preterm infant with Early onset SCN2A developmental and epileptic encephalopathy

Supplementary Information

Supplementary Figures

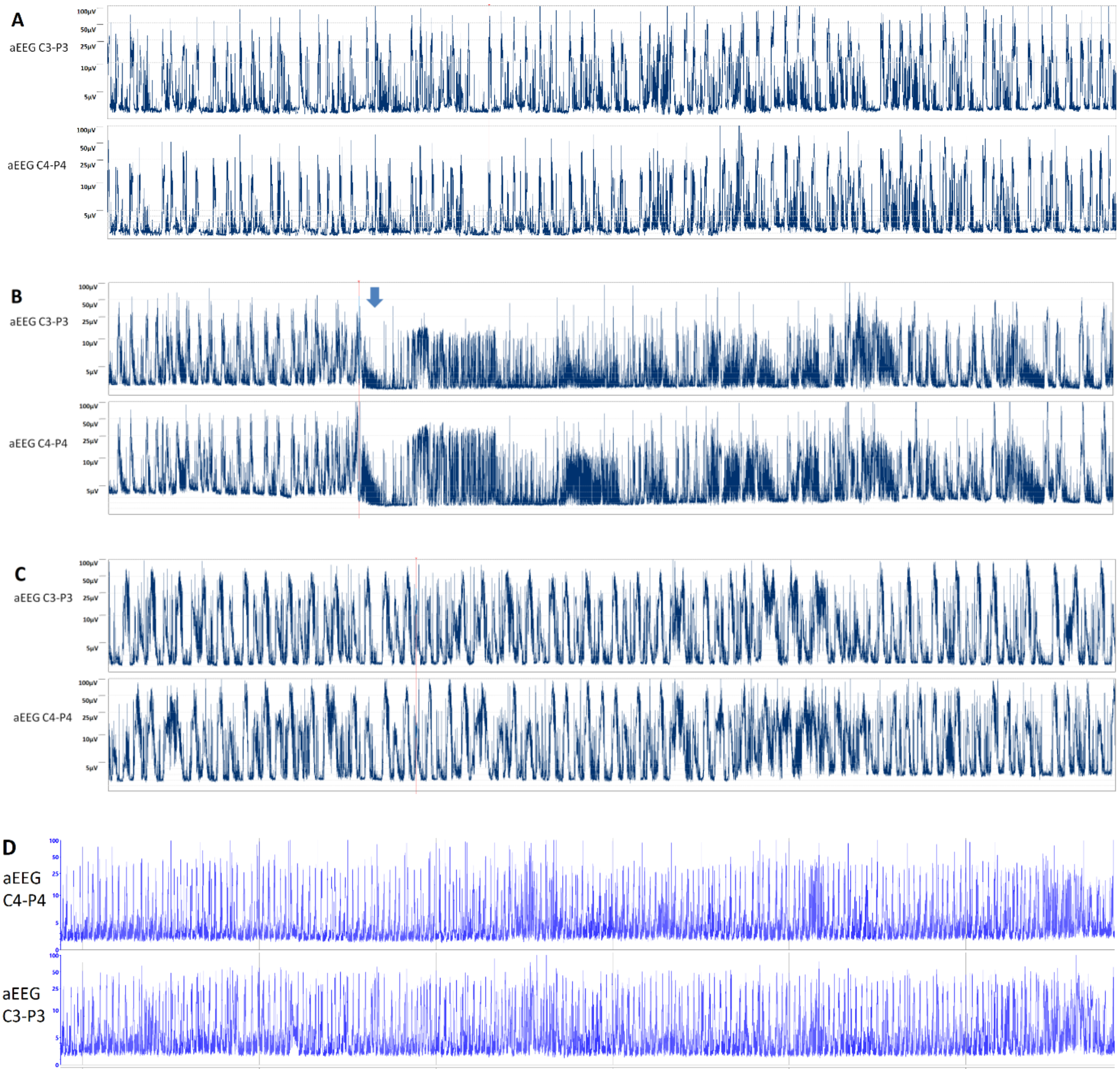

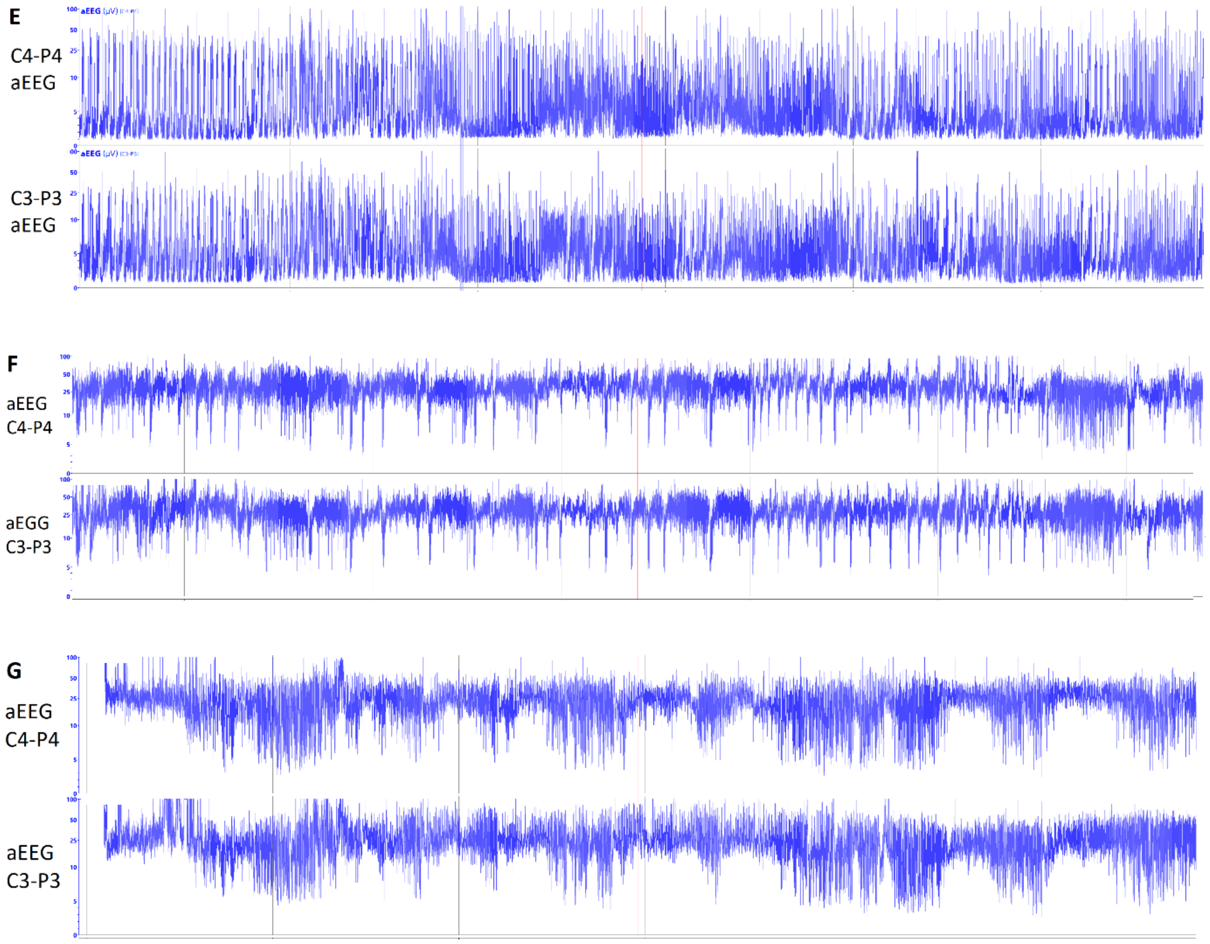

Supplementary Figure 1A-G: Patient´s aEEG recordings. All aEEG traces comprise 6h of recording).

A-D prior to PRAX-222 administration, **E-G:** after / under ongoing PRAX-222 administration. **A** Representative aEEG trace showing a typical sawtooth pattern resembling EEG-status in week one. **B** Seizure reduction after several loading doses of phenytoin (arrow) with peak serum levels of phenytoin >40µg/ml in week three. **C** Seizure reduction was not sustainable as status pattern reoccurred even with phenytoin levels >40µg/ml. Seizures were subclinical or motor seizures. **D** Week seven (1 day before first ASO administration) showing sawtooth pattern resembling EEG-status epilepticus. **E-G** Modulation and further reduction of seizure activity (often in close timely relationship to SCB administration) 1, 7 and 10 weeks after first administration of PRAX-222.

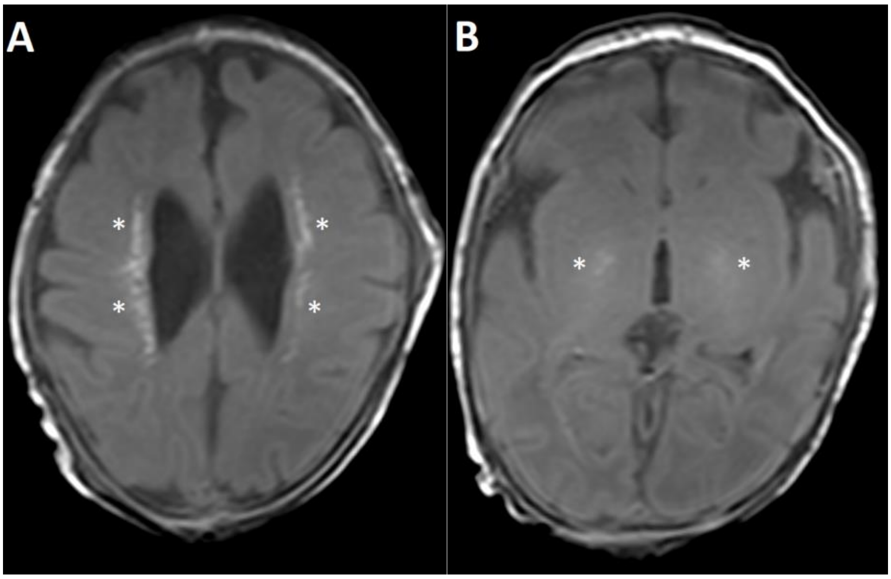

Supplementary Figure 2: postnatal brain MRI at the age of 5 weeks shows T1 hyperintensities and periventricular thinning.

T1 weighted image shows T1w bilateral symmetric hyperintensities with blooming artifacts in the periventricular medullary bed (**A**) and basal ganglia (**B**). Hyperintensities are marked with an asterisk.

A (age: 1 month)

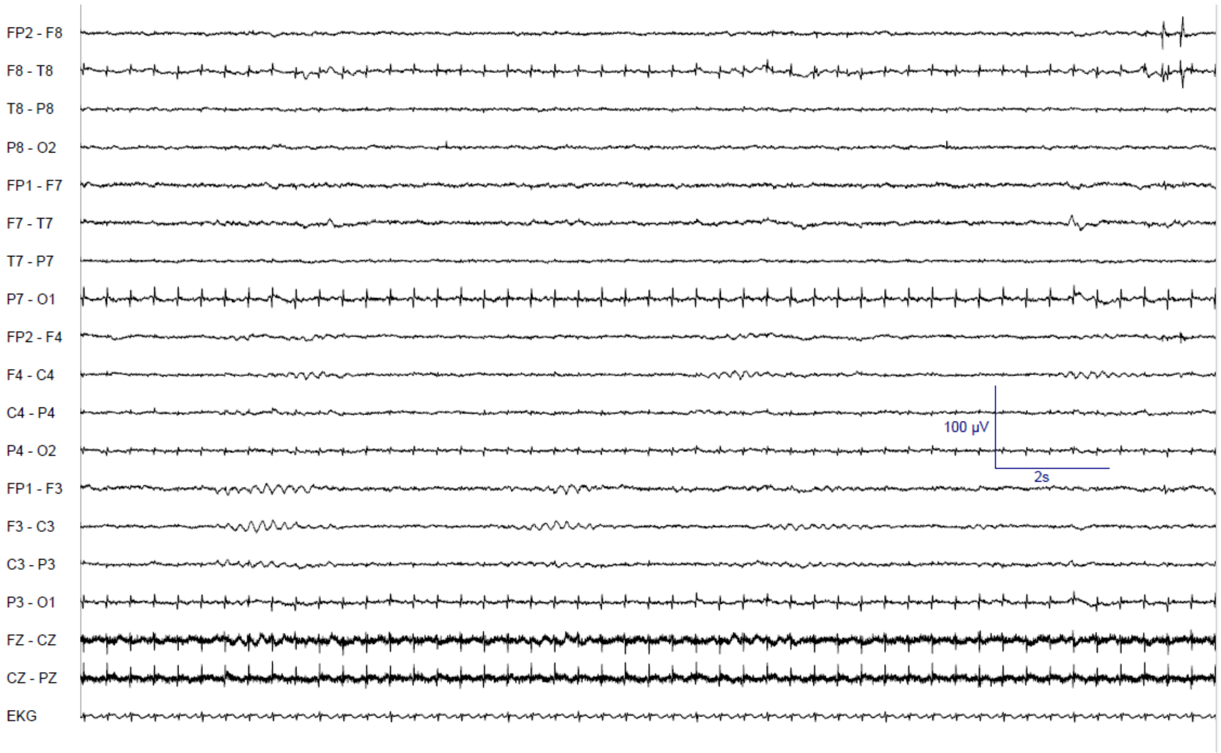

B (age: 2 months)

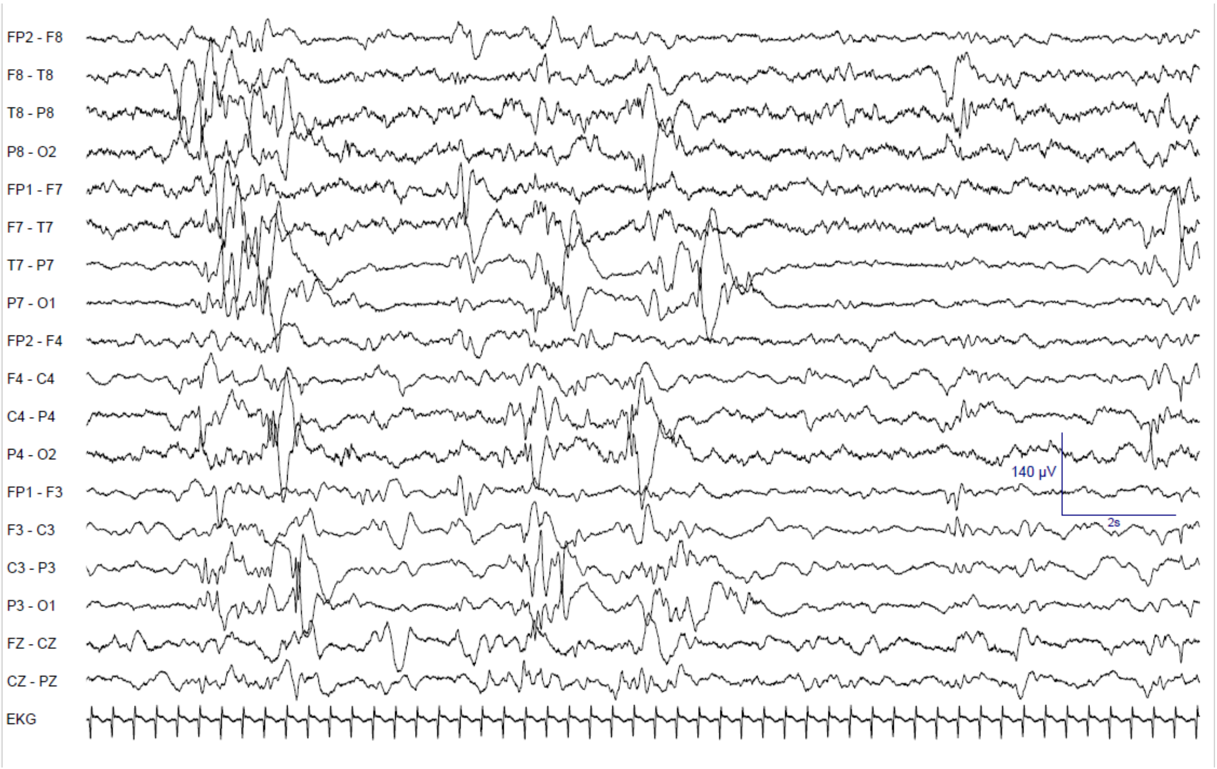

C (age: 3 months)

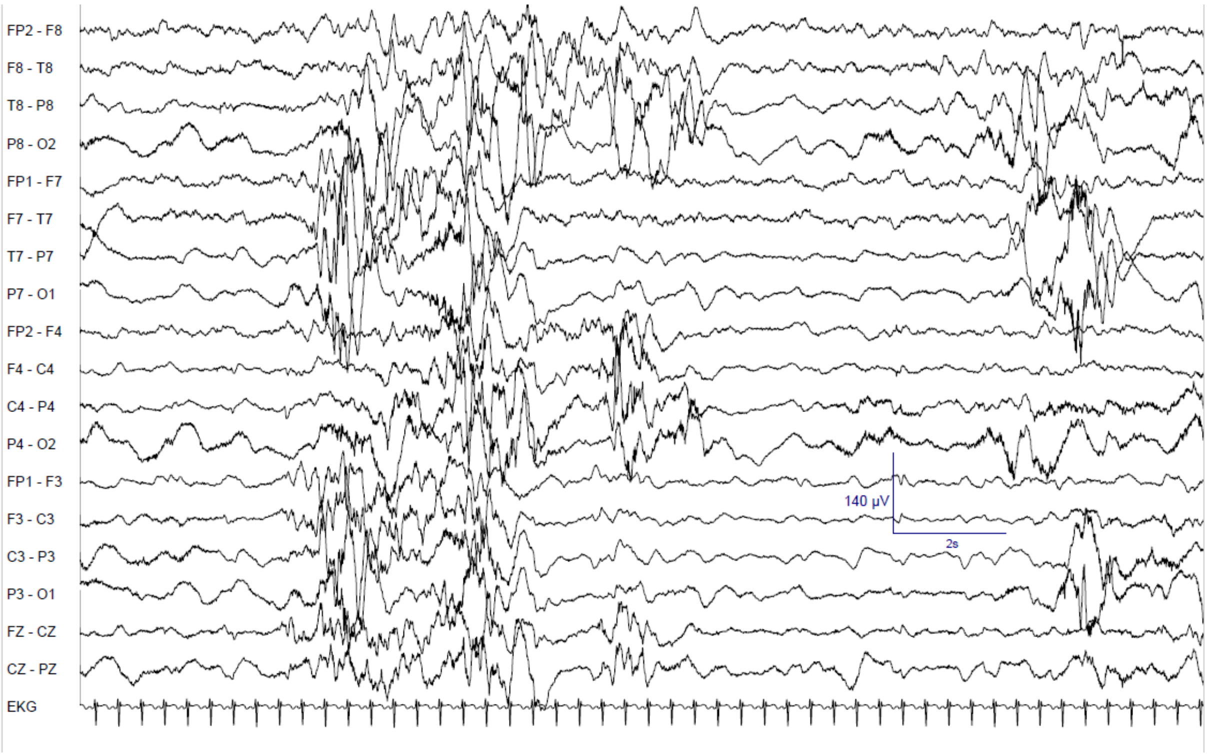

D (age: 4 months)

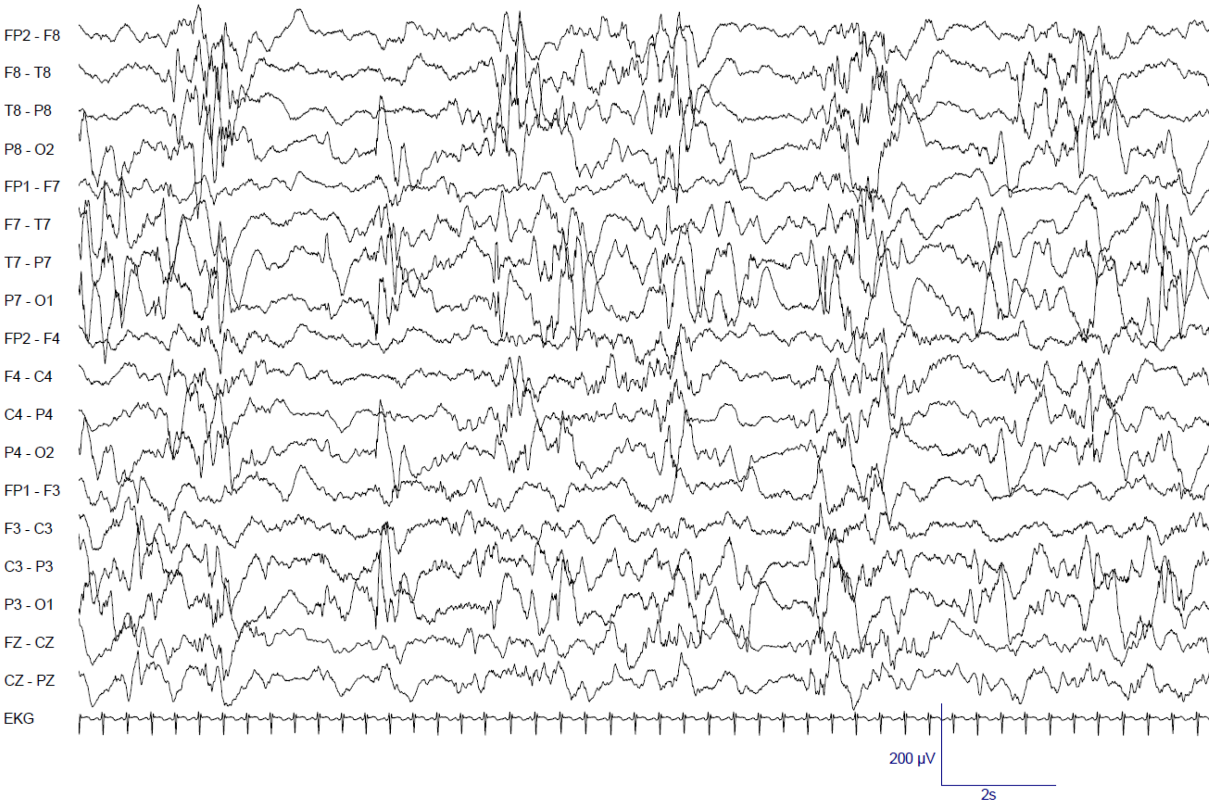

E (age: 5 months)

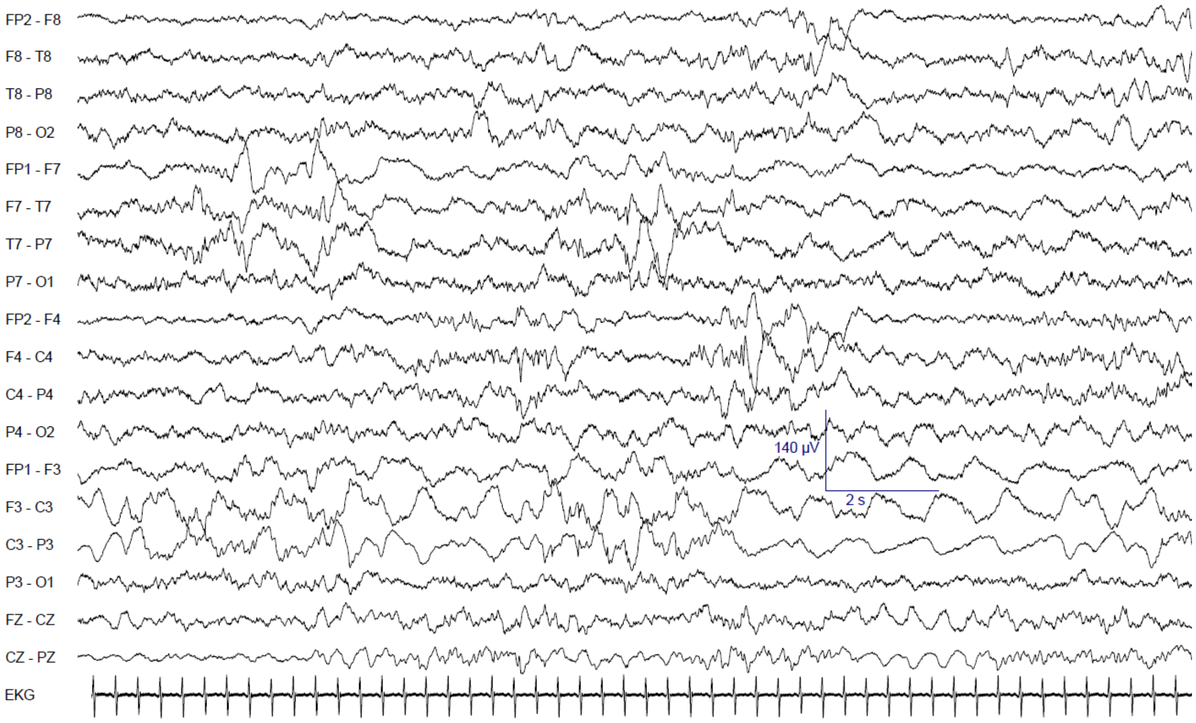

F (age: 9 months)

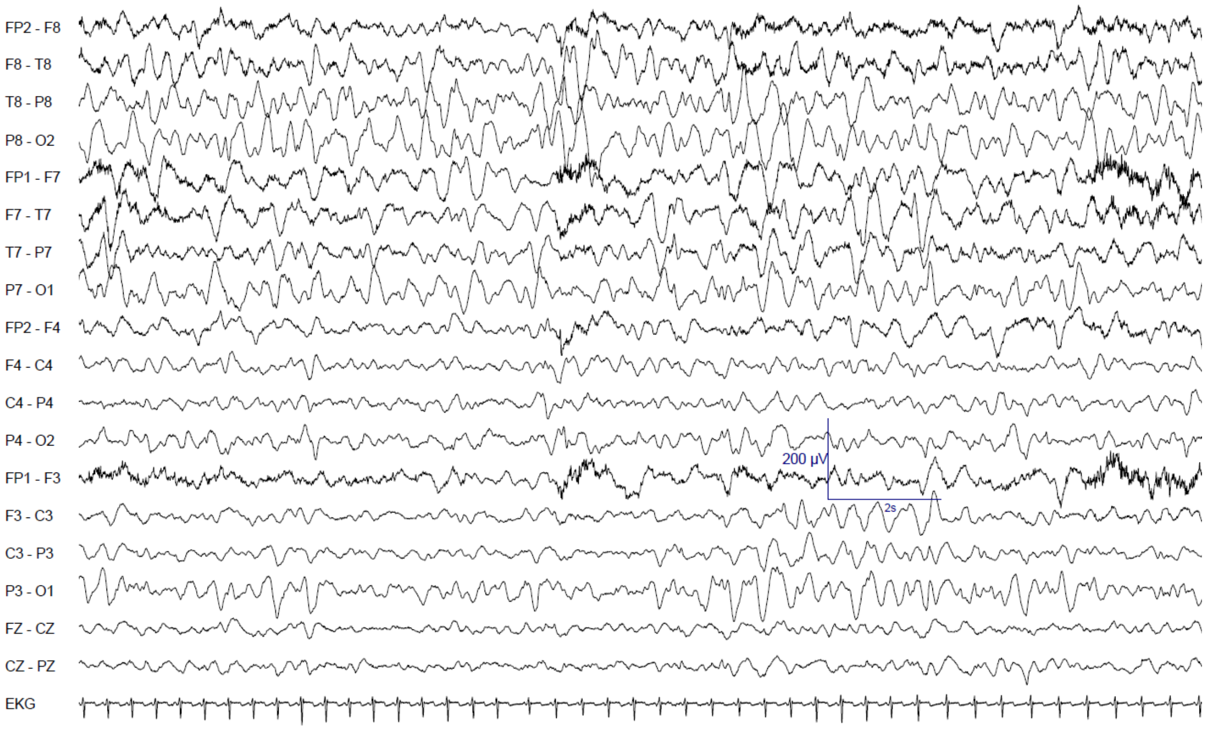

G (age: 10 months)

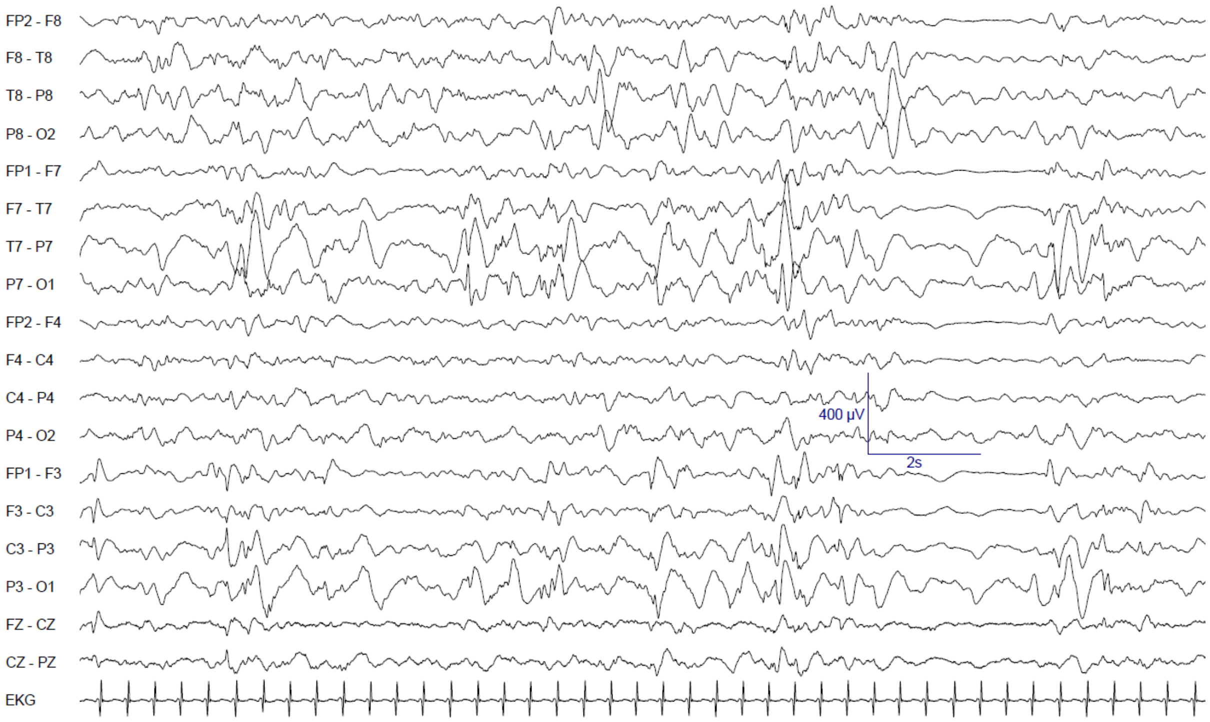

H (age: 13 months)

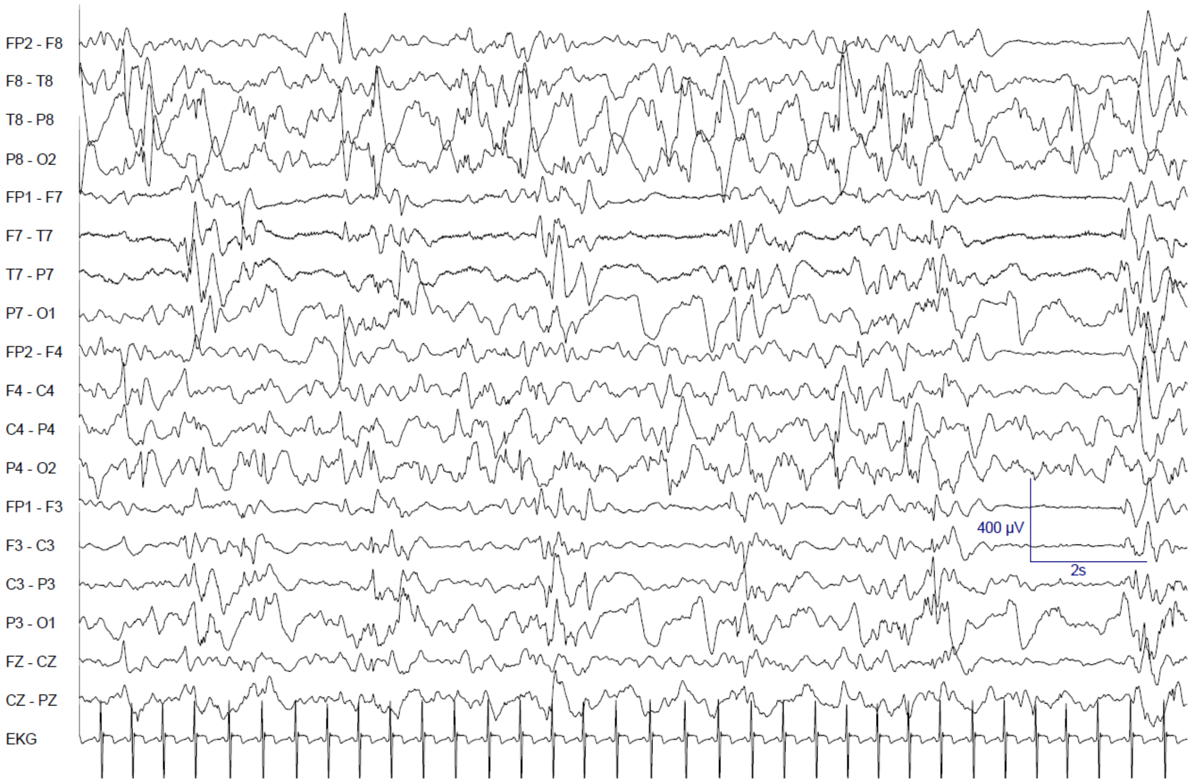

I (age: 14 months)

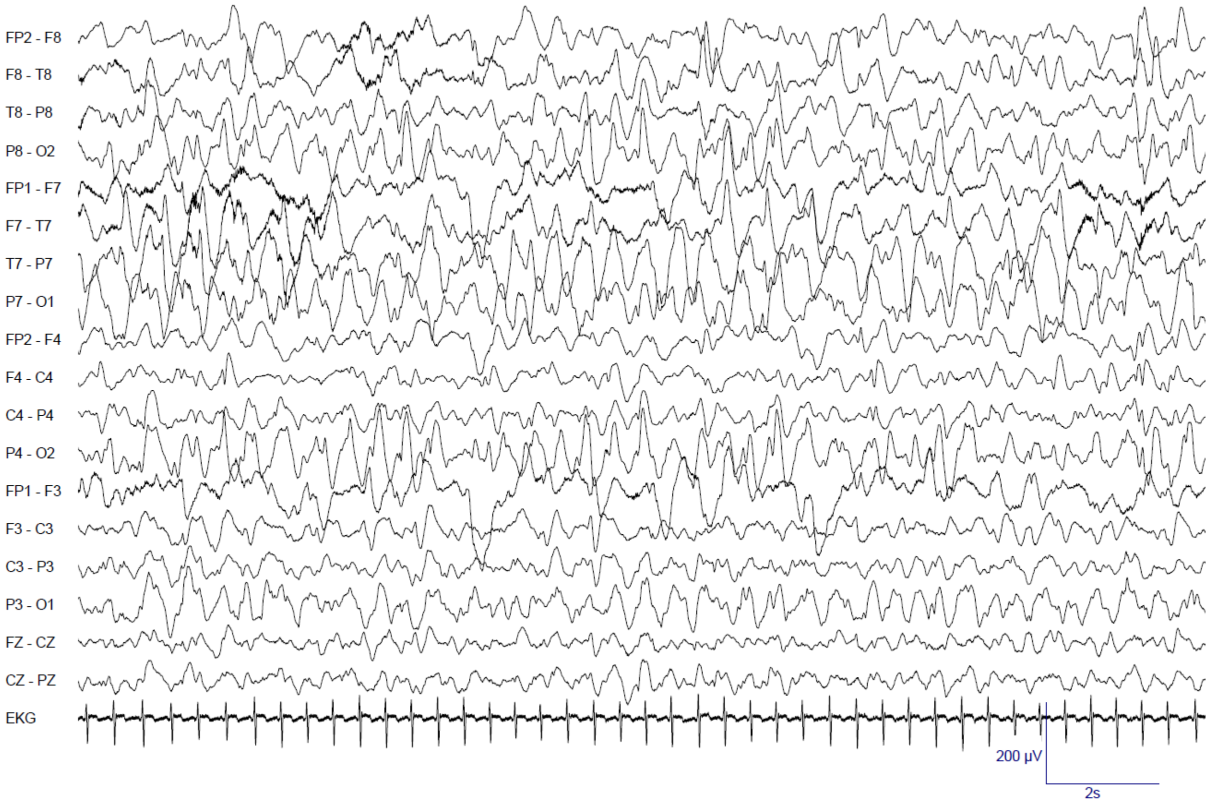

J (age: 15 months)

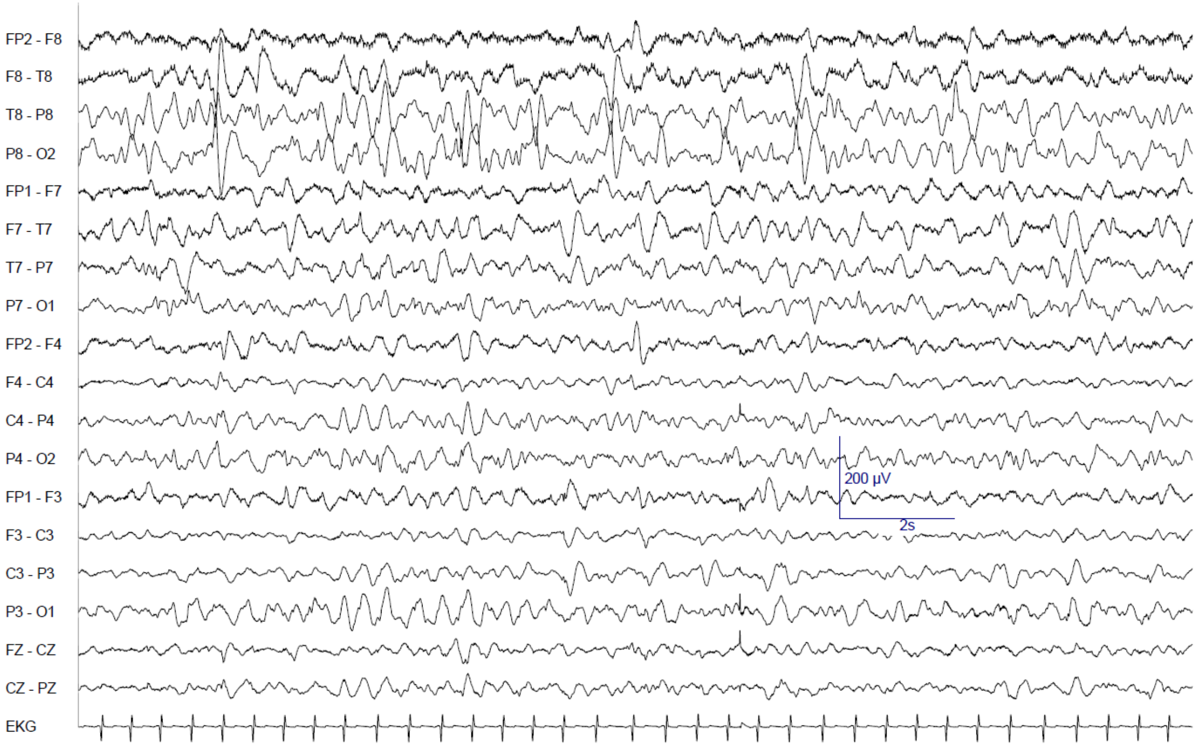

K (age: 16 months)

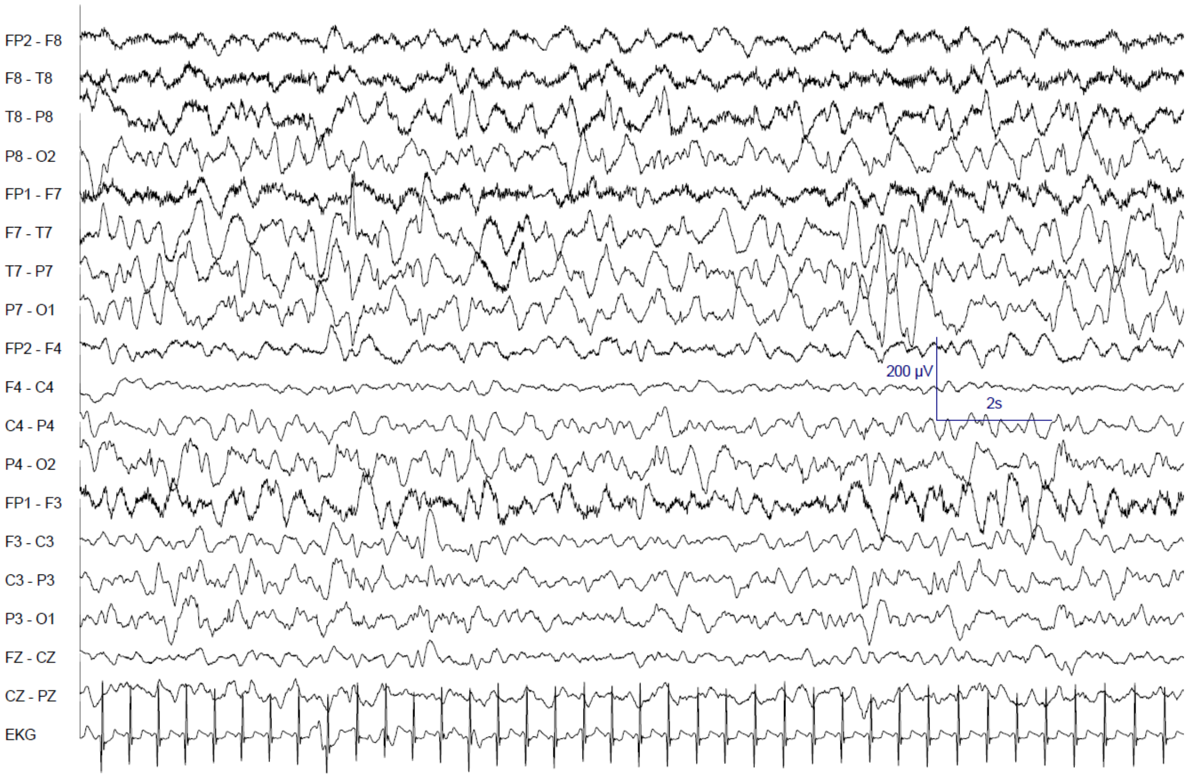

L (age: 17 months)

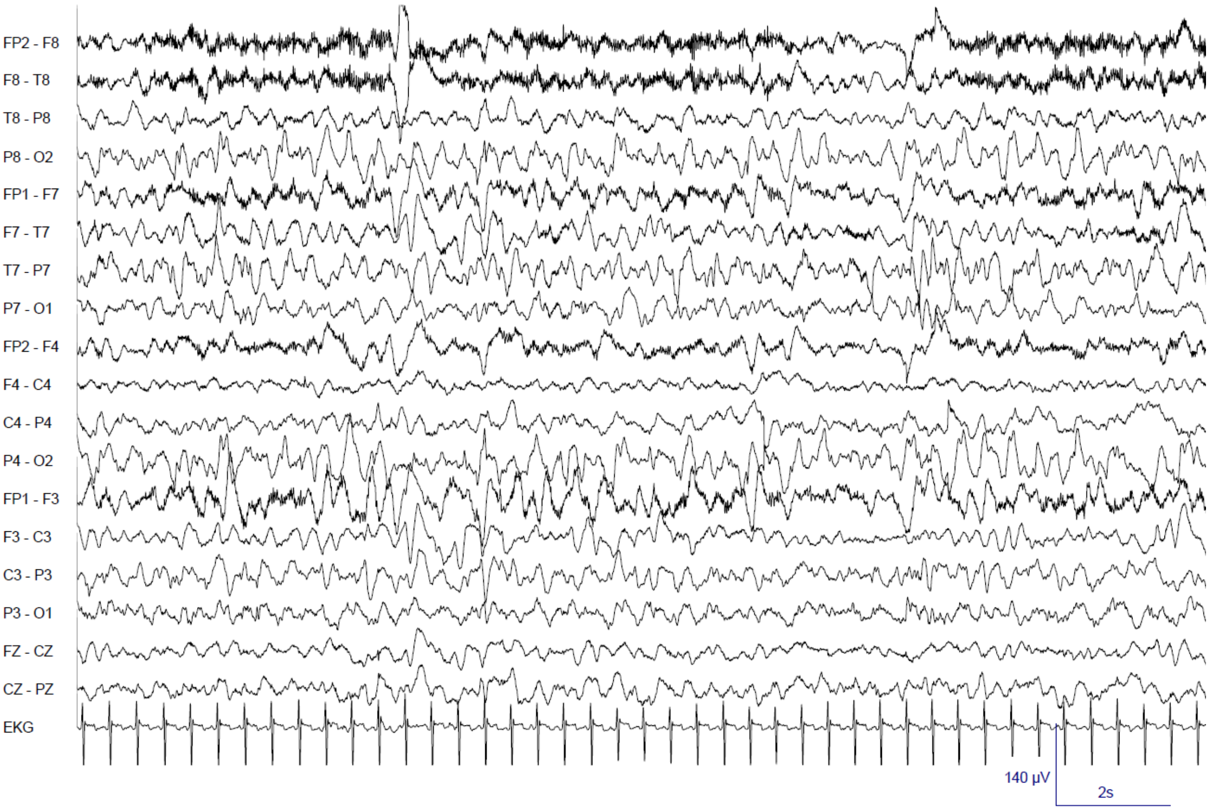

M (age: 19 months)

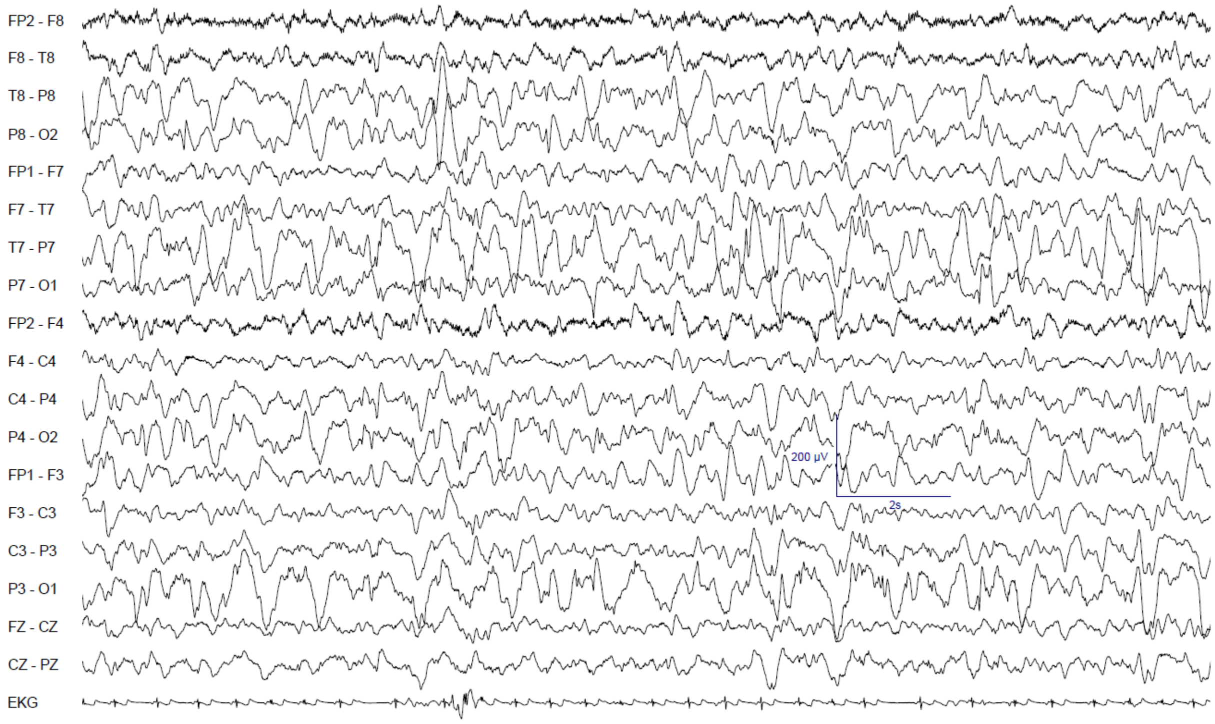

N (age: 20 months)

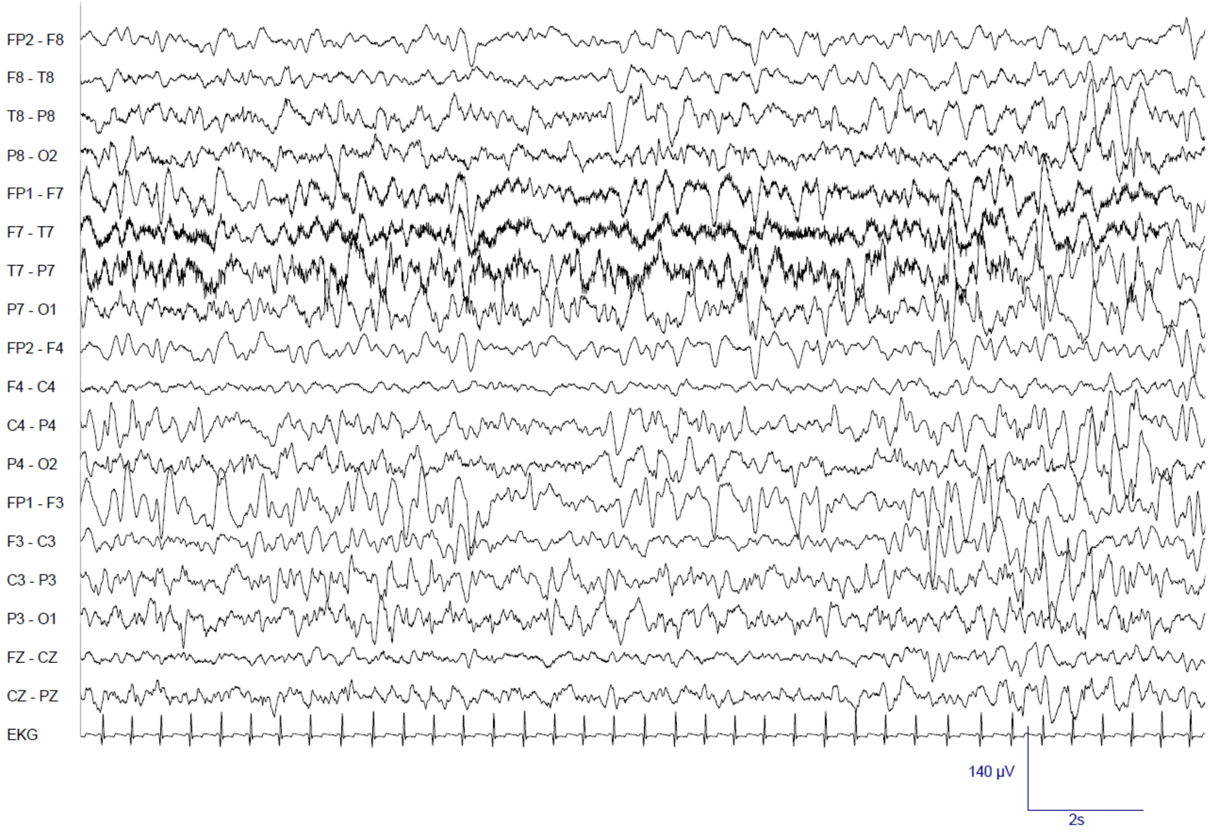

O (age: 21 months)

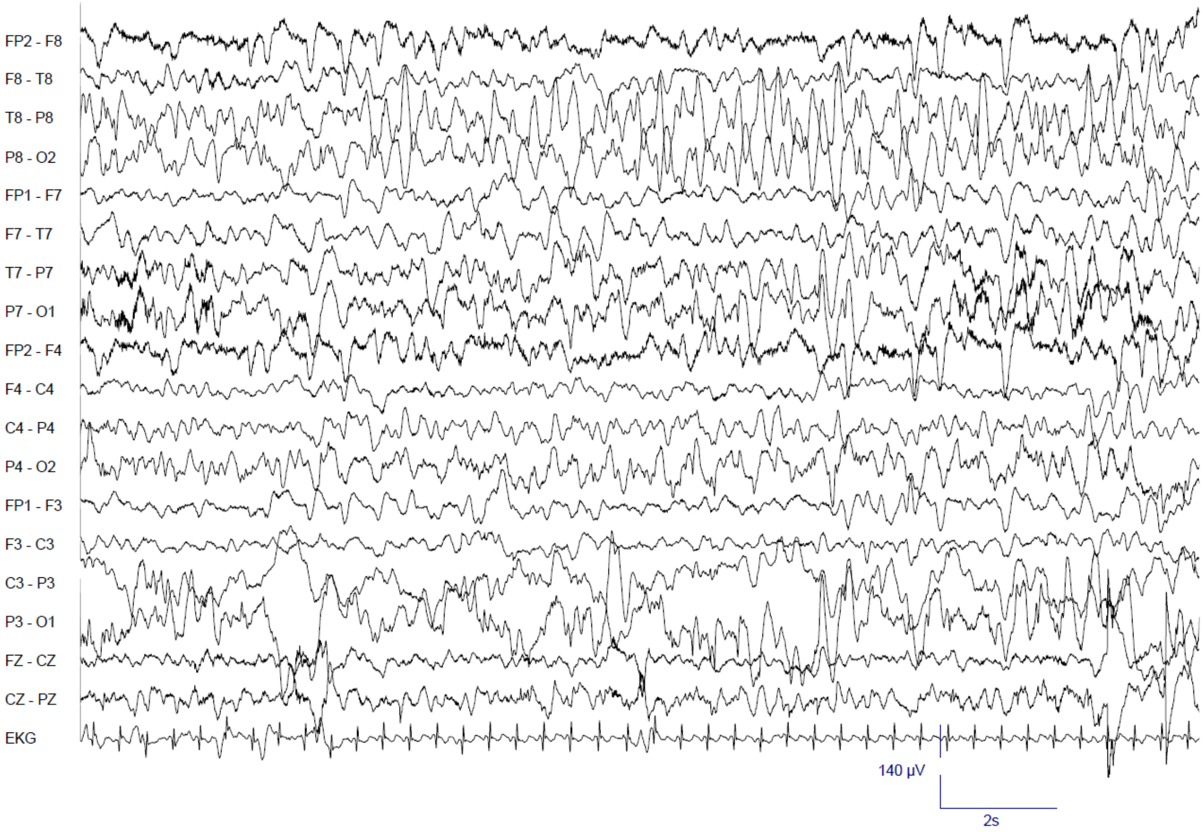

Supplementary Figure 3: Continuous evaluation of sleep-wake EEGs. Repetetive interictal sleep-wake EEGs from at the ages of 1 (A), 2 (B), 3 (C), 4 (D), 5 (E), 9 (F), 10 (G), 13 (H), 14 (I), 15 (J), 16 (K), 17 (L), 19 (M), 20 (N) and 21 (O) months shows signs of an epileptic encephalopathy with multiregional epileptic discharges. Over the course of the disease, EEGs show signs of brain maturation with discontinuous patterns only until the age of 13 months and no worsening of interictal EEGs upon treatment.

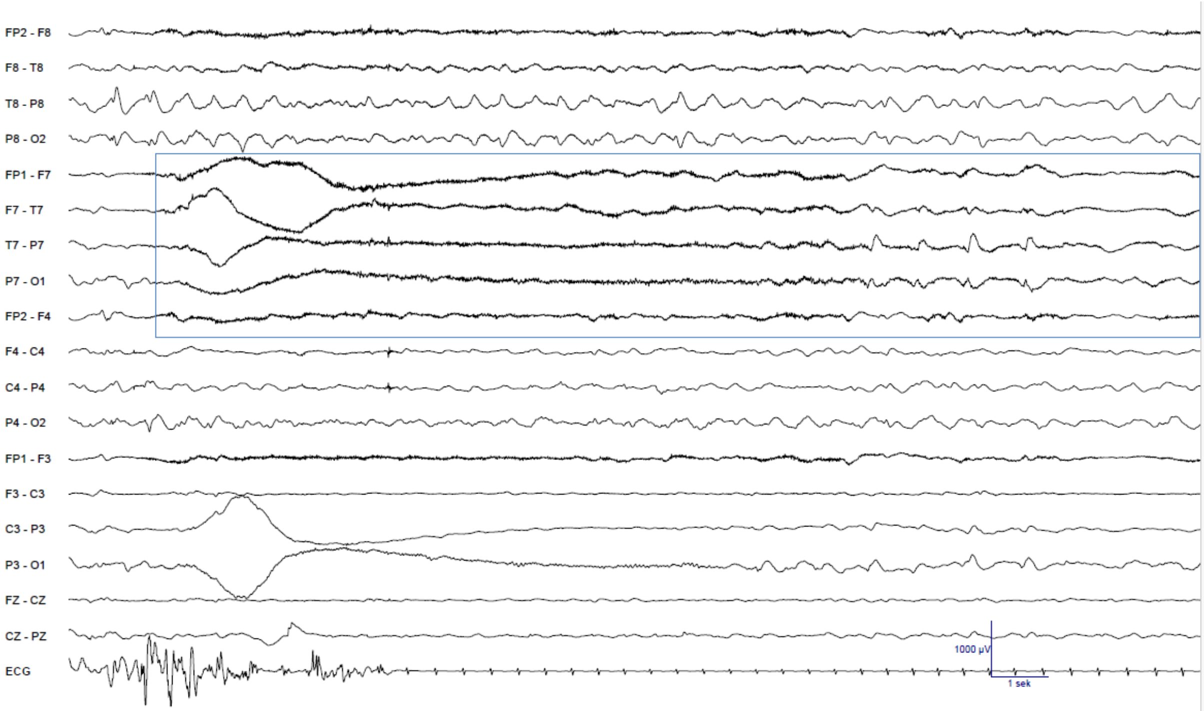

Supplementary Figure 4: Ictal EEG at the age of 22 months. EEG seizure pattern corresponding to Supplementary Video 1: Paroxysmal rapid activity predominantly over the left-sided leads (blue box)

Supplementary Tables

Supplementary Table 1. Biophysical parameters of wild-type (WT) and A1329D $Na_V1.2$ channel variants

Biophysical property	WT	A1329D	P value
Current density at −10 mV (pA/pF)<br>n	−479.7 ± 72.2<br>14	−481.0 ± 100.9^{NS}<br>12	0.991
Activation<br>$V_{0.5,act}$ (mV)<br>k_{act} (mV)<br>n	 −17.26 ± 0.49<br>6.97 ± 0.22<br>14	 −16.66 ± 0.40^{NS}<br>7.17 ± 0.21 ^{NS}<br>12	 0.364<br>0.689
Inactivation<br>$V_{0.5,inact}$ (mV)<br>k_{inact} (mV)<br>n	 −51.84 ± 0.26<br>7.40 ± 0.25<br>14	 −45.83 ± 0.27****<br>6.54 ± 0.27*<br>12	 <0.0001<br>0.028
Persistent I_{Na} (% of total)<br>at −25 mV<br>at −10 mV<br>at +5 mV<br>at +15 mV<br>n	 1.25 ± 0.34<br>0.87 ± 0.39<br>0.86 ± 0.39<br>0.27 ± 0.50<br>14	 4.45 ± 0.71*<br>5.33 ± 0.49****<br>5.15 ± 0.55****<br>3.48 ± 0.87*<br>12	 0.012<br><0.0001<br><0.0001<br>0.012
Time course of fast inactivation (ms)<br>τ_f at −35 mV<br>τ_f at −30 mV<br>τ_f at −25 mV<br>n	 3.14 ± 0.36<br>2.65 ± 0.47<br>2.04 ± 0.27<br>14	 6.32 ± 0.52****<br>4.52 ± 0.32****<br>2.87 ± 0.12*<br>12	 <0.0001<br><0.0001<br>0.040
Time course of recovery[§] (ms)<br>τ<br>n	 1.34 ± 0.07<br>14	 1.19 ± 0.04^{NS}<br>12	 0.088

Data are represented as mean±SEM; n, number of cells measured; $V_{0.5,(in)act}$, membrane potential for half-maximal (in)activation; $k_{(in)act}$, slope factor of steady-state (in)activation

curve; τ, time constant; τ_f, fast time constant; $^{*}P < 0.05$, $^{****}P < 0.0001$ compared with WT. §Recovery from fast inactivation.

Supplementary Table 2. Seizure Frequency within the extended observational period.

Age (months)	Seizures/hour	ASM serum concentrations	
		CBZ [µg/ml]	PHE [µg/ml]
9	3	2,4	24
10	2	5	25
11	5	5,3	13
12		5,8	10
13	4	10,7	8
14	4	8,4	0
15	4	10,1	
16	4		
17	5		
18	4	11	

Seizure frequency and ASM serum concentrations during longterm follow up between 9 and 8 months of age. ASM: anti-seizure medication, PHE: phenytoine, CBZ: carbamazepine

Supplementary Table 3. Action potential frequencies in the AIS hybrid model incorporating wild-type (WT) or A1329D $Na_V1.2$ conductance.

Stimulus current (pA)	Action potential frequency (Hz)						
	WT			A1329D			P value
	Mean	SEM	n	Mean	SEM	n	
-2	0	0	12	0	0	10	>0.9999
0	0	0	12	0	0	10	>0.9999
2	0	0	12	0	0	10	>0.9999
4	0.07	0.071	12	0.50	0.801	10	>0.9999
6	0.35	0.225	12	15.9	2.907	10	<0.0001
8	2.71	0.881	12	27.9	1.666	10	<0.0001
10	6.55	1.294	12	32.6	1.220	10	<0.0001
12	4.78	1.127	12	36.6	0.926	10	<0.0001
14	3.64	0.400	12	38.9	0.833	10	<0.0001
16	2.92	0.286	12	33.2	3.349	10	<0.0001
18	2.42	0.227	12	25.5	5.329	10	<0.0001
20	2.21	0.186	12	19.4	5.852	10	<0.0001
22	2.07	0.177	12	13.6	4.903	10	0.0004
24	1.56	0.144	12	8.5	2.395	10	0.1443

Abbreviations: n, number of cells; $Na_V1.2$, voltage-gated sodium channel type 1.2; pA, picoamps; SEM, standard error of the mean; Mean action potential frequency values of WT and A1329D variants in each row were compared using two-way ANOVA with Sidak's multiple comparison test, resulting in the p values shown.
